# Whole-Genome Sequence of *Bacillus* sp. SDLI1, Isolated from the Social Bee *Scaptotrigona depilis*

**DOI:** 10.1128/genomeA.00174-16

**Published:** 2016-03-24

**Authors:** Camila R. Paludo, Antonio C. Ruzzini, Eduardo A. Silva-Junior, Gleb Pishchany, Cameron R. Currie, Fábio S. Nascimento, Roberto G. Kolter, Jon Clardy, Mônica T. Pupo

**Affiliations:** a School of Pharmaceutical Sciences of Ribeirão Preto, University of São Paulo, Ribeirão Preto, São Paulo, Brazil; b Department of Biological Chemistry and Molecular Pharmacology, Harvard Medical School, Boston, Massachusetts, USA; c Department of Microbiology and Immunobiology, Harvard Medical School, Boston, Massachusetts, USA; d Department of Bacteriology, University of Wisconsin, Madison, Wisconsin, USA; e Department of Biology, FFCLRP, University of São Paulo, Ribeirão Preto, São Paulo, Brazil

## Abstract

We announce the complete genome sequence of *Bacillus* sp. strain SDLI1, isolated from larval gut of the stingless bee *Scaptotrigona depilis*. The 4.13-Mb circular chromosome harbors biosynthetic gene clusters for the production of antimicrobial compounds.

## GENOME ANNOUNCEMENT

A number of *Bacillus* spp. engage in symbiotic relationships with solitary and social bees (1–4). These bacteria can improve insect nutrition (4) and in some instances provide protection against entomopathogens through small-molecule chemical defenses (5, 6). A recent report of a mutualistic relationship between the social bee *Scaptotrigona depilis* and a fungus (7) highlighted the importance of studying bee-associated microbiota. Here, we report a high-quality genome sequence for *Bacillus* sp. SDLI1 from the larval gut of the Brazilian stingless bee *S. depilis*.

The bacterium named *Bacillus* sp. SDLI1 was isolated from *S. depilis* larvae that were subjected to surface sterilization (8). Briefly, the insects were submerged in 70% ethanol for 2.5 min, transferred to 5% sodium hypochlorite for 5 min, then transferred to 70% ethanol for 1 min, and washed with sterile water. The lacerated larvae were inoculated in International *Streptomyces* Project medium number 2 agar plates, then incubated at 30°C.

Genomic DNA was extracted from an overnight monoculture of *Bacillus* sp. SDLI1 in lysogeny broth (Lennox, Sigma-Aldrich) using the Wizard genomic DNA purification kit (Promega). A long insert (15- to 20-kb) DNA library was prepared and sequenced at the Duke Center for Genomic and Computational Biology Sequencing and Genomic Technologies Shared Resource core facility. Two single-molecule real-time (SMRT) cells (9) were run on a PacBio RSII instrument using P6-C4 chemistry. The Hierarchical Genome Assembly Process (10) generated a single 4,143,589-bp polished contig with a mean coverage of 338× and mean quality value of 48.5. Manual inspection of the contig revealed that the termini were degenerate, and one end was removed to produce the final circular assembly of 4,134,042 bp with a G-C content of 45.8%.

A cursory inspection of four *Bacillus* sp*.* SDLI1 housekeeping genes (*dnaJ*, *rpoB*, *gyrA*, and *recA*) revealed that it is a member of the *B. subtilis* group. A comparison of the complete nucleotide sequences of chromosomes within this group suggests that *Bacillus* sp. SDLI1 is most closely related to *Bacillus siamensis* KCTC 13613^T^ (82.7%, GenBank accession no. AJVF00000000.1) and *B. siamensis* XY18 (82.6%, GenBank accession no. LAGT00000000.1), based on *in silico* genome-to-genome distance calculation and reporting the DNA-DNA hybridization value from formula 2 as a metric (11). Genome annotation using RAST (12) predicted 4,298 coding sequences, including 19 rRNA genes and 86 tRNA genes. Finally, antiSMASH (13) analyses indicated that *Bacillus* sp. SDLI1 genome encodes biosynthetic gene clusters with high sequence identity to those that produce fengycin, bacillomycin, bacillaene, macrolactin, surfactin, bacilysin, bacillibactin, and difficidin.

### Nucleotide sequence accession number.

The complete genome sequence has been deposited to the NCBI under the accession number CP013950.
